# Testing and comparing two self-care-related instruments among older Chinese adults

**DOI:** 10.1371/journal.pone.0182792

**Published:** 2017-08-09

**Authors:** Lina Guo, Ulrika Söderhamn, Jacqueline McCallum, Xianfei Ding, Han Gao, Qiyun Guo, Kun Liu, Yanjin Liu

**Affiliations:** 1 Department of Neurology, the first Affiliated Hospital of Zhengzhou University, Zhengzhou, Henan, People's Republic of China; 2 Faculty of Health and Sport Sciences, Department of Health and Nursing Sciences, University of Agder, Grimstad, Aust-Agder, Southern Norway; 3 School of Health and Life Sciences, Department of Nursing & Community Health, Glasgow Caledonian University, Glasgow, Scotland, United Kingdom; 4 Department of Integrated ICU, the first Affiliated Hospital of Zhengzhou University, Zhengzhou, Henan, People's Republic of China; 5 School of Nursing, Jinzhou Medical University, Jinzhou, Liaoning, People's Republic of China; 6 Department of Nursing, the first Affiliated Hospital of Zhengzhou University, Zhengzhou, Henan, People’s Republic of China; Universidad Maimonides Facultad de Ciencias de la Salud, ARGENTINA

## Abstract

**Objectives:**

The study aimed to test and compare the reliability and validity, including sensitivity and specificity of the two self-care-related instruments, the Self-care Ability Scale for the Elderly (SASE), and the Appraisal of Self-care Agency Scale-Revised (ASAS-R), among older adults in the Chinese context.

**Methods:**

A cross-sectional design was used to conduct this study. The sample consisted of 1152 older adults. Data were collected by a questionnaire including the Chinese version of SASE (SASE-CHI), the Chinese version of ASAS-R (ASAS-R-CHI) and the Exercise of Self-Care Agency scale (ESCA). Homogeneity and stability, content, construct and concurrent validity, and sensitivity and specificity were assessed.

**Results:**

The Cronbach's alpha (α) of SASE-CHI was 0.89, the item-to-total correlations ranged from r = 0.15 to r = 0.81, and the test-retest correlation coefficient (intra-class correlation coefficient, ICC) was 0.99 (95% CI, 0.99–1.00; P<0.001). The Cronbach's α of ASAS-R-CHI was 0.78, the item-to-total correlations ranged from r = 0.20 to r = 0.65, and the test-retest ICC was 0.95 (95% CI, 0.92–0.96; P<0.001). The content validity index (CVI) of SASE-CHI and ASAS-R-CHI was 0.96 and 0.97, respectively. The findings of exploratory and confirmatory factor analyses (EFA and CFA) confirmed a good construct validity of SASE-CHI and ASAS-R-CHI. The Pearson's rank correlation coefficients, as a measure of concurrent validity, between total score of SASE-CHI and ESCA and ASAS-R-CHI and ESCA were assessed to 0.65 (*P*<0.001) and 0.62 (P<0.001), respectively. Regarding ESCA as the criterion, the area under the receiver operator characteristic (ROC) curve for the cut-point of SASE-CHI and ASAS-R-CHI were 0.93 (95% CI, 0.91–0.94) and 0.83 (95% CI, 0.80–0.86), respectively.

**Conclusion:**

There is no significant difference between the two instruments. Each has its own characteristics, but SASE-CHI is more suitable for older adults. The key point is that the users can choose the most appropriate scale according to the specific situation.

## Introduction

With the largest population in the world, as well as having the fastest population growth, it is estimated that China's elderly population will reach more than 400 million by 2016. However, China is a developing country, and the "no-rich aging first" condition has lead to an unsound system of pension insurance, which causes stress for older adults [1,2].

With the development of the economy, and the transformation of society and its impact on the change of family structure, the number of older adults who live alone or with a spouse is rapidly increasing. The families of older adults who are single or live with a spouse account for 25% of all families, and it is predicted that this will increase to 90% by 2030 [3].

With the decline of physiological function, the morbidity rate of the older population is 3–5 times the rest of the population, particularly due to chronic diseases. These diseases result in the decline of the physical and mental well-being of the elderly. This is occurring at the same time as self-care is declining. Yet, older adults are wishing to live in their own homes if possible. Therefore, the challenge which older adults face is not only disease and ill-health, but also the problems the daily life causes [4,5,6].

The goal put forward in the field of public health in the 21st century is to achieve gradual improvement of health in the aging population, including promotion of health, prevention of disease, improving self-care facilities, whilst improving quality of life, and extending life expectancy. The contradiction between "the healthy aging" and "the serious situation" gets more pronounced with the rapid growth of the aging population, and the increasing number of older adults who are alone or have diseases. All those have aroused widespread concern in society [7,8,9]. This leads to the question of how the problems confronting older adults can be addressed against this background?

Self-care can be defined as the practice of activities that individuals initiate on their own behalf in maintaining health and well-being. Self-care refers to the ability of self-care activities or self-management and it is influenced by many factors, such as age, level of development, life experiences, cultural background, and health status. The self-care of individuals can change depending on different development stages and different health conditions [10]. According to Sousa et al. [11] older adults through positive self-care can not only enhance health-promoting behavior, but also improve the specific capability for those chronically ill through self-management [11]. In addition, improving the self-management of older adults can save national resources, help older adults to live up to their potential, improve the quality of life and realize self-value. As a result, improving the ability of self-care of the older adults is an important way to realize "healthy aging", and it is also a suitable model for the medical services of China [2].

The progress of assessment tools regarding self-care ability is conducive to further promotion of self-care. Correctly, evaluating the self-care ability of older adults is helpful to develop targeted measures to improve their self-care ability. This requires dedicated instruments to measure the self-care ability of this special group. Currently, many scales are used to assess the self-care ability of older adults, such as the Exercise of Self-care Agency scale (ESCA) [12,13], the Appraisal of Self-care Agency Scale (ASAS) [14,15], the Self-care Ability Scale for the Elderly (SASE) [16,17,18], Lorensen's Self-Care Ability Scale (LSCS) [19] and Elderly People's Scale for Self-care Capacity Classifying (ESCC) [20]. The most widely used among these instruments are the ASAS and its revised version, ASAS-R [13,21–25]. But at present, only ESCA scale is commonly used to evaluate self-care ability of older adults in Mainland China.

To enrich and develop instruments for assessing self-care ability of the older adults in China, we translated, according to recommend procedures [9,26], the ASAS-R and SASE into Simplified Chinese, and made them more suitable for assessing self-care and self-care ability, respectively, of older adults in China [27,28]. The Chinese version of SASE (SASE-CHI) [28] and ASAS-R (ASAS-R-CHI) [27] have been confirmed as valid and reliable instruments, with simple content, clear dimension and easy to understand. It is, however, of importance to further test and compare the characteristics and the application effect of the two self-care instruments to verify suitable instruments for use among older Chinese people.

### Aim and objective

The study aimed to test and compare the reliability and validity, including sensitivity and specificity of the two self-care-related instruments, the SASE, and the ASAS-R, among older adults in the Chinese context.

## Methods

### Study design

A cross-sectional survey was conducted among older adults who were selected from the community and hospitals in Jinzhou, a city in the northeast part of People’s Republic of China.

### Sample

This study was approved by the Ethical Review Board of Jinzhou Medical University, Jinzhou, China. Written informed consent was obtained from all study participants after they received information about the study and before the data collection interview took place [29].

The study sample consisted of 1152 older adults, from three districts and two hospitals in Jinzhou city from April to October 2014. The inclusion criteria were: (1) ≥60 years old, (2) able to communicate verbally in Chinese, (3) have no cognitive disorder (≥26 scores of the Montreal Cognitive Assessment (MoCA). (MoCA is a quick and easy measure of cognitive functioning that has been widely used to screen patients with mild cognitive impairments in clinical evaluation and research. A higher score manifests a better cognitive function, and ≥26 scores indicate a normal cognition [30].), (4) willing to take part in the study, and (5) living in Jinzhou more than six months.

The sample size of 1,152 older adults exceeded published criteria for psychometric analyses such as 20:1 subjects to number of scale items ratio [31] or the specification of N >1,000 as "excellent" [32]. It reflects the thinking of Osborne and Costello [33] that in conducting psychometric research, a large sample is required. In addition, recruitment of both community-dwelling and hospitalized older adults was purposeful with the intention of obtaining a diverse sample with chronic conditions.

### Data collection

Prior to conducting the survey, a preliminary investigation was carried out amongst 100 older adults (70 home-dwelling persons and 30 hospital patients), to identify any possible problems in understanding the questions in the questionnaire.

Survey data were collected at community stations where older adults often held activities, such as parks, squares, or venues where committee meetings took place. There were eight community stations within three districts, the older adults came to these survey stations voluntarily (*n* = 622). Hospital surveys were conducted in medical wards, geriatric wards, and rehabilitation centers in two hospitals (*n* = 530).

Data were collected during one-to-one, face-to-face interviews with trained investigators who assisted participants with completing the study questionnaire by presenting items in a neutral, matter-of-fact way to avoid introducing response bias.

After two weeks of the investigation, 86 older adults (52 home-dwelling persons and 34 hospital patients) were randomly selected from the total participants with contact information, to test the test-retest reliability.

### Measurements

The questionnaire included demographic variables such as age, gender, height, weight, body mass index (BMI), marital status, educational level, alcohol drinking habits, smoking habits, health insurance, and chronic diseases, contact information, and the three instruments SASE-CHI, ASAS-R-CHI, and ESCA.

#### SASE-CHI

Söderhamn et al. [16] designed SASE in 1996. It is a five-point Likert scale that consists of 17 items distributed on three factors, factor 1 (the repertoire, 8 items), factor 2 (the environment, 2 items), and factor 3 (the goals, 7 items). Four items are stated negatively. Every item ranges from 5 to 1 between “totally agree” to “totally disagree”. The possible total score ranges between 17 and 85. A higher total score indicates higher self-care ability. The SASE has already been tested and used in Sweden and Norway [6,16,17,18,25,34,35].

#### ASAS-R-CHI

ASAS-R is the revised version of the ASAS. The ASAS was improved by Evers and other scholars in 1986 [14], based on Orem’s self-care deficit theory, with 24 items. It emphasizes the ability of self-care behaviors, observation, judgment, decision-making and implementation. This scale has been used and validated in several countries, including America, the Netherlands, Norway, Switzerland, Mexico, and Hong Kong, China [11]. Despite its widespread use, some authors have considered that the original version has a complex factor structure and some items are not included in any factors [11]. Sousa et al. [11] have altered the original version into ASAS-R, a 15-item, 3-factor version scale; factor 1 (having power for self-care, 6 items), factor 2 (developing power for self-care, 5 items) and factor 3 (lacking power for self-care, 4 items) [11]. ASAS-R is a five-point Likert scale ranging from 1 to 5 between “totally disagree” and “totally agree”. Four items are negatively expressed. A higher total score indicates higher self-care ability, and maximum possible score is 75 [11,24]. The ASAS-R also has a Brazilian version [24].

#### ESCA

The Americans Kearny and Fleischer [12] designed ESCA according to Orem’s self-care deficit theory, in 1979. It was translated into Chinese and revised by Wang and Laffrey [13] in 2000, with internal consistency estimates ranging from 0.86 to 0.92 and test-retest reliability of 0.91 after one week [13]. Evidence for construct validity was demonstrated by significant correlations in the expected directions with personal resources and perceived health [13]. The scale is the most widely used measure in mainland China [36–38]. The Cronbach's α was 0.89 in the present study. ESCA aims to assess the ability of the older adults with 43 items distributed on four factors, factor 1 (motivation, 9 items), factor 2 (knowledge base, 9 items), factor 3 (active vs. passive response to situations, 12 items) and factor 4 (sense of self-worth, 13 items). The total score of ESCA ranges from 0 to 172, the higher the score, the higher the self-care. If the score is more than 66% of the total score (≥116points), it indicates a high level of self-care, and less than 66% (<116 points) of the total score indicates a middle-low level [12,13].

### Translation procedure

The translation process of the SASE and ASAS-R into Simplified Chinese consisted of several steps. (1) A bilingual professional translator translated the two scales from English into Chinese, then another bilingual professional translator translated the translated Chinese versions back into English. The two translators worked separately [26]. (2) A group of bilingual persons, including three nursing experts and two psychology experts, examined the original English versions and the back translated scales to resolve discrepancies in the meaning of the scale items and to evaluate the cultural and the linguistic equivalence of each item. (3) A preliminary field test was conducted among 10 older adults with the trial Chinese versions, and modifications were made according to the participants’ feedback on the items, until a consensus was reached for the final SASE-CHI and ASAS-R-CHI in terms of wording, clarity, and cultural equivalence.

### Data analysis

SPSS 21.0 and AMOS 17.0 were used to analyze the data. Cronbach's alpha (α) of the SASE-CHI and ASAS-R-CHI, the Guttman Split-Coefficient, Cronbach's α of their respective factors and item-to-total correlations (estimated by Pearson's rank correlation coefficients) were used to test the homogeneity of the scales. The stability (test-retest reliability) of SASE-CHI and ASAS-R-CHI was assessed by calculating two-way intra-class correlation coefficients (ICC) for absolute agreement with 95% confidence intervals from 86 participants' two total scores of SASE-CHI and ASAS-R-CHI, respectively.

To assess the content validity index (CVI) of ASAS-R-CHI and SASE-CHI, six specialists were invited to assess content validity according to four levels; 4 = highly relevant, 3 = quite relevant, 2 = somewhat relevant, and 1 = not relevant. The ''highly relevant'' and "quite relevant" choices yielded a score of 1, and "somewhat relevant " and "not relevant" choices yielded a score of 0.

Exploratory and confirmatory factor analyses (EFA and CFA) were used to examine the construct validity of the SASE-CHI and ASAS-R-CHI, with the total sample randomly split into two groups. One group of 576 participants was used to conduct the EFA, and the other group of 576 was used for CFA.

Concurrent validity was estimated by Pearson's rank correlation coefficients between total score of SASE-CHI and ESCA and their factors, and between total score of ASAS-R-CHI and ESCA and their factors.

Receiver operator characteristic (ROC) curves, sensitivity, specificity, positive predictive value (PPV) and negative predictive value (NPV), and the Youden's index were estimated to find suitable cut-off points of SASE-CHI and ASAS-R-CHI, respectively. The ESCA was regarded as the criterion. The total score ≥116 of ESCA indicates a high level of self-care; and the total score <116 of ESCA indicates a middle-low level of self-care [12,13].

## Results

### Sample

Considering ESCA as a good standard, 869 participants were defined with a middle-low level of self-care, and 283 participants were defined with a high level of self-care. The characteristics of the sample are presented in Table 1.

**Table 1:** Characteristics of the sample(*n* = 1152).

Variables	Total sample *n* = 1152	Community-dwelling *n* = 622	Hospitals *n* = 530
**Age in years (Mean ± SD)**	71.69±8.13	70.65±6.53	72.91±9.54
≥60 years old (*n*/%)	523(45.4)	286(46.0)	237(44.7)
≥70 years old (*n*/%)	397(34.5)	258(41.5)	139(26.2)
≥80 years old (n/%)	206(17.8)	78(12.5)	128(24.1)
≥90 years old (n/%)	26(2.3)	0(0.0)	26(5.0)
**Gender (*n*/%)**			
Male	613(53.2)	325(52.3)	288(54.3)
Female	539(46.8)	297(47.7)	242(45.7)
**BMI(Mean ± SD)**	20.61±2.58	21.38±2.47	19.80±2.39
**Marital status (*n*/%)**			
Single	4(0.3)	2(0.3)	2(0.4)
Married	829(72.0)	483(77.7)	346(65.3)
Divorced	7(0.6)	5(0.8)	2(0.4)
Widowed	299(26.0)	129(20.7)	170(31.9)
No response	13(1.1)	3(0.5)	10(2.0)
**Education (*n*/%)**			
≤9 years	663(57.6)	378(60.8)	285(53.8)
>9years and ≤12 years	286(24.8)	143(23.0)	143(27.0)
>12 years	203(17.6)	101(16.2)	102(19.2)
**Smoking habits (*n*/%)**			
Yes	926(80.4)	490(78.8)	436(82.3)
No	226(19.6)	132(21.2)	94(17.7)
**Alcohol drinking habits (*n*/%)**			
Yes	920(79.8)	479(77.0)	441(83.2)
No	232(20.2)	143(23.0)	89(16.8)
**Health insurance (*n*/%)**			
Yes	1123(97.5)	611(98.2)	512(96.6)
No	29(2.5)	11(1.8)	18(3.4)
**Chronic diseases (*n*/%)**			
Yes	650(56.42)	209(33.6)	441(83.2)
No	502(43.58)	413(66.4)	89(16.8)

BMI: body mass index.

### Reliability

#### Homogeneity

The Cronbach's α of SASE-CHI was 0.89, the Guttman Split-Coefficient of SASE-CHI was 0.86, and the three factors of SASE-CHI yielded a Cronbach's α of 0.86, 0.71 and 0.80, respectively. The Cronbach's α of ASAS-R-CHI was 0.78, the Guttman Split-Coefficient of ASAS-R-CHI was 0.72, and the three factors of ASAS-R-CHI yielded a Cronbach's α of 0.77, 0.72 and 0.71, respectively.

There was no single item that if deleted would improve the overall Cronbach's α for SASE-CHI (Table 2) or ASAS-R-CHI (Table 3). The item-to-total correlations of SASE-CHI ranged between r = 0.15 and r = 0.81, where the average correlation was r = 0.59 (Table 2), and the item-to-total correlations of ASAS-R-CHI ranged between r = 0.20 and r = 0.65, where the average correlation was r = 0.50 (Table 3).

**Table 2:** Item-to-total score Pearson's rank correlation coefficients for SASE-CHI (*n* = 1152).

Items	*r*	Cronbach's α if item deleted
S1 I want to be able to go to places that are not within walking distance.	0.60^※^	0.88
S2 I can in a satisfying way maintain my personal hygiene.	0.72^※^	0.88
S3 I can in a satisfying way clean my mouth and my teeth (dentures).	0.81^※^	0.87
S4 I can manage to do my own housekeeping.	0.81^※^	0.87
S5 I can manage my own daily shopping.	0.81^※^	0.87
S6 I don't feel safe when I move in my environment.	0.15	0.89
S7 I can change things in my life in order to enhance my state of health.	0.25^※^	0.87
S8 I can move around enough to feel good.	0.69^※^	0.89
S9 I know what I have to do in order to feel safe in my environment.	0.49^※^	0.88
S10 I feel satisfied in my life.	0.65^※^	0.88
S11 I want to manage to do my own daily shopping	0.62^※^	0.88
S12 I want to manage to be alone.	0.44^※^	0.89
S13 I want to manage to do my own housekeeping.	0.54^※^	0.89
S14 I don't know how much strength I have.	0.54^※^	0.88
S15 I can influence my life in such a way that I find it satisfying.	0.60^※^	0.88
S16 I don't manage to be alone.	0.52^※^	0.88
S17 I don't manage to dress and undress myself.	0.71^※^	0.88
The average item-to-total score coefficients of SASE-CHI	0.59	0.89

*Note*. SASE-CHI: the Chinese version of the Self-Care Ability Scale for the Elderly; S1-S17: Item 1-Item 17 of SASE-CHI.

^※^P<0.01.

**Table 3:** Item-to-total score Pearson's rank correlation coefficients for ASAS-R-CHI (*n* = 1152).

Items	*r*	Cronbach's α if item deleted
A1 As circumstances change, I make the needed adjustments to stay healthy.	0.58^※^	0.77
A2 If my mobility is decreased, I make the needed adjustments.	0.58^※^	0.76
A3 When needed, I set new priorities in the measures that I take to stay healthy.	0.63^※^	0.76
A4 I often lack energy to care for myself in the way that I know I should.	0.60^※^	0.76
A5 I look for better ways to take for myself.	0.65^※^	0.76
A6 When needed, I manage to take time to care for myself.	0.65^※^	0.76
A7 If I take a new medication, I obtain information about the side effects to better care for myself.	0.56^※^	0.78
A8 In the past, I have changed some of my old habits in order to improve my health.	0.20^※^	0.78
A 9 I routinely take measures to insure the safety of myself and my family.	0.61^※^	0.76
A10 I regularly evaluate the effectiveness of things that I do to stay healthy.	0.50^※^	0.77
A11 In my daily activities I seldom take time to care for myself.	0.27^※^	0.78
A12 I am able to get information I need, when health is threatened.	0.61^※^	0.76
A13 I seek help when unable to care for myself.	0.29^※^	0.78
A14 I seldom have time for myself.	0.31^※^	0.78
A15 I am not always able to care for myself in a way I would like.	0.42^※^	0.78
The average item-to-total score coefficients of ASAS-R-CHI	0.50	0.78

*Note*. ASAS-R-CHI: the Chinese version of the revised form of the Appraisal of Self-care Agency Scale; A1- A 15: Item 1-Item 15 of ASAS-R-CHI.

^※^P<0.01.

#### Stability

The test-retest between the twice total score of SASE-CHI provided an ICC of 0.99 (95% CI, 0.99–1.00; *P*<0.001), and the test-retest between twice total score of ASAS-R-CHI provided an ICC of 0.95 (95% CI, 0.92–0.96; *P*<0.001).

### Validity

#### Content validity

The CVI, yielded a value of 0.96 for SASE-CHI, and a value of 0.97 for ASAS-R-CHI.

#### Construct validity

**Fig 1:**
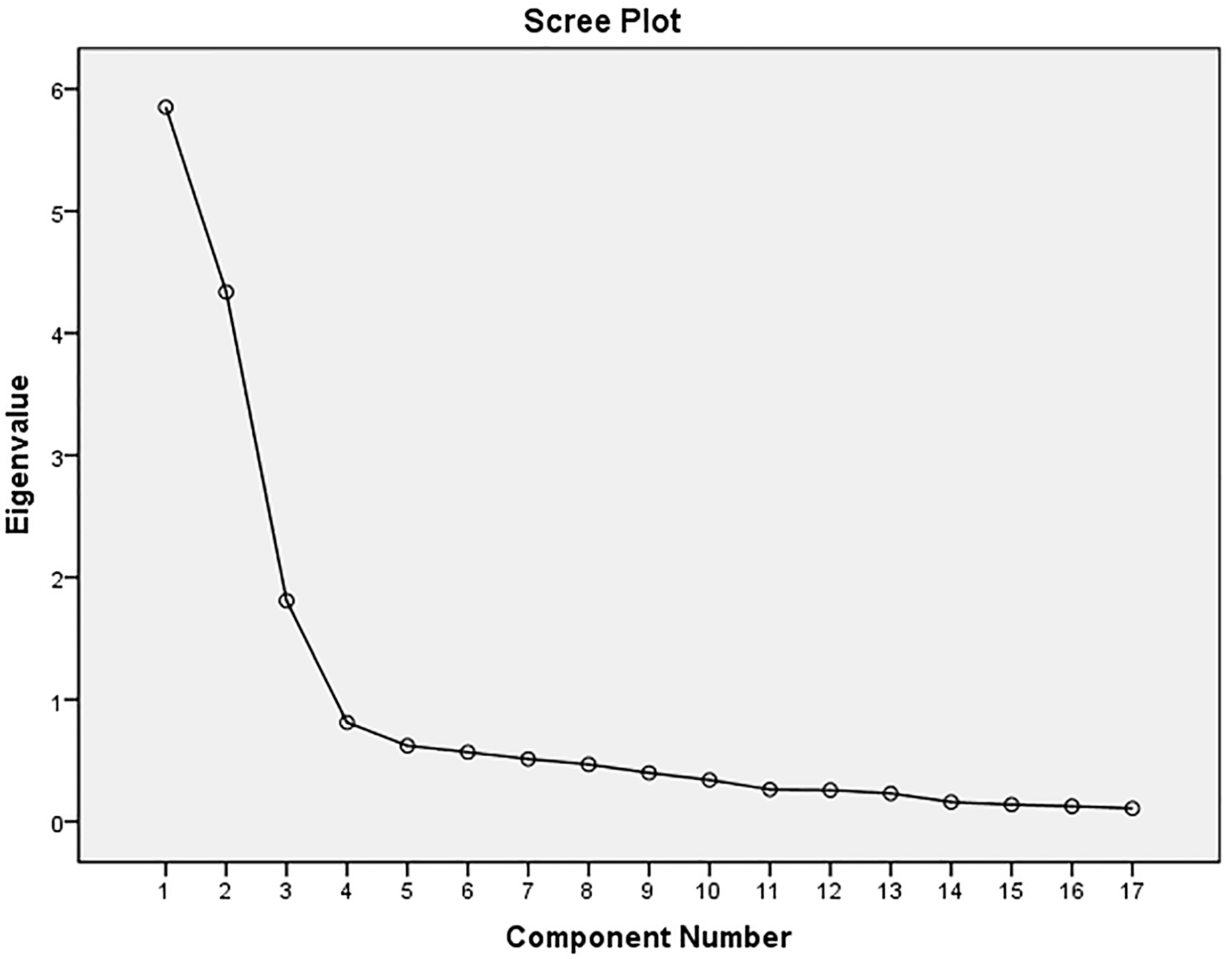
Scree Plot of SASE-CHI (*n* = 576). *Note*. SASE-CHI: the Chinese version of Self-care Ability Scale for the Elderly.

**Fig 2:**
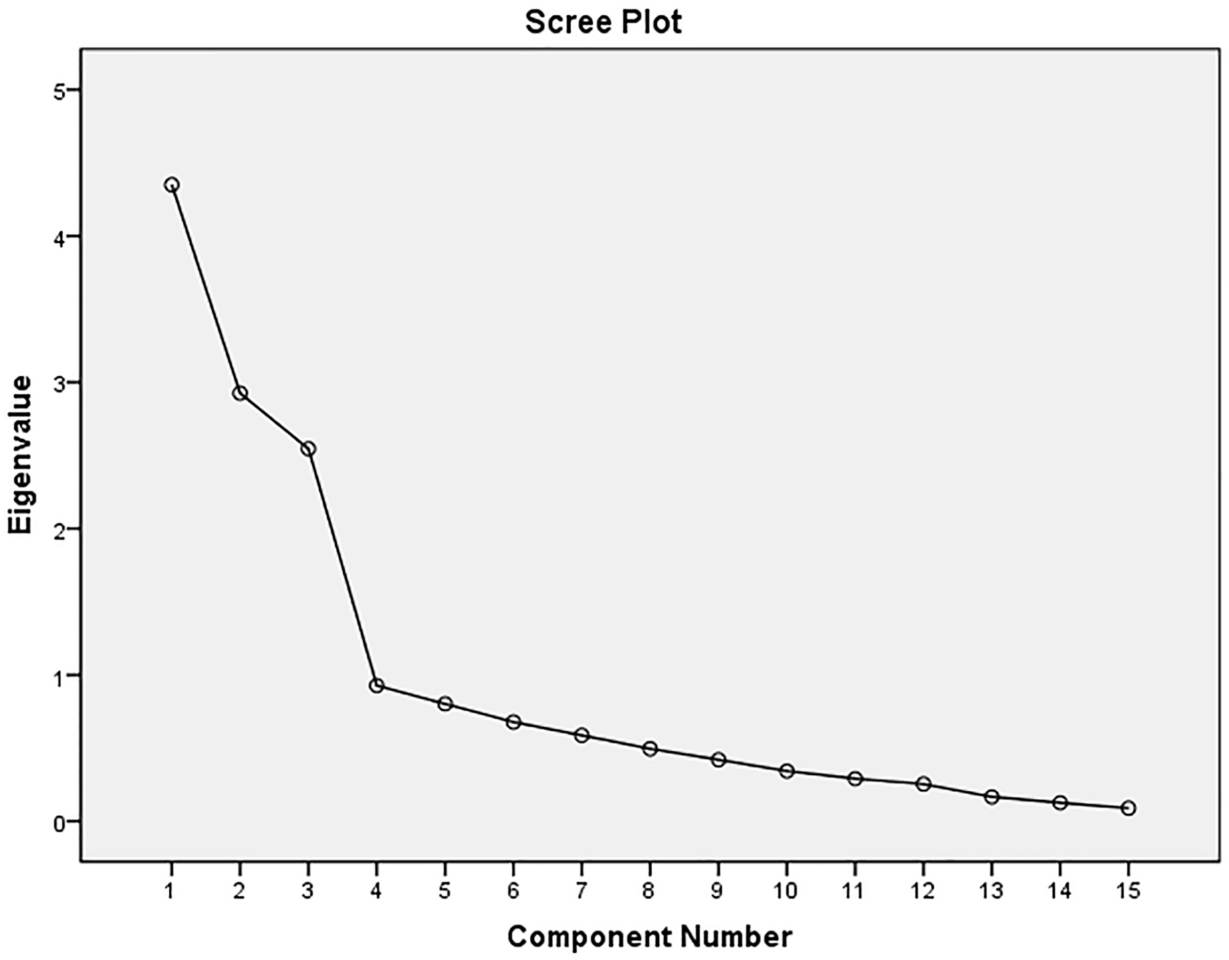
Scree Plot of ASAS-R-CHI (*n* = 576). *Note*. ASAS-R -CHI: the Chinese version of the revised form of the Appraisal of Self-care Agency Scale.

**Fig 3:**
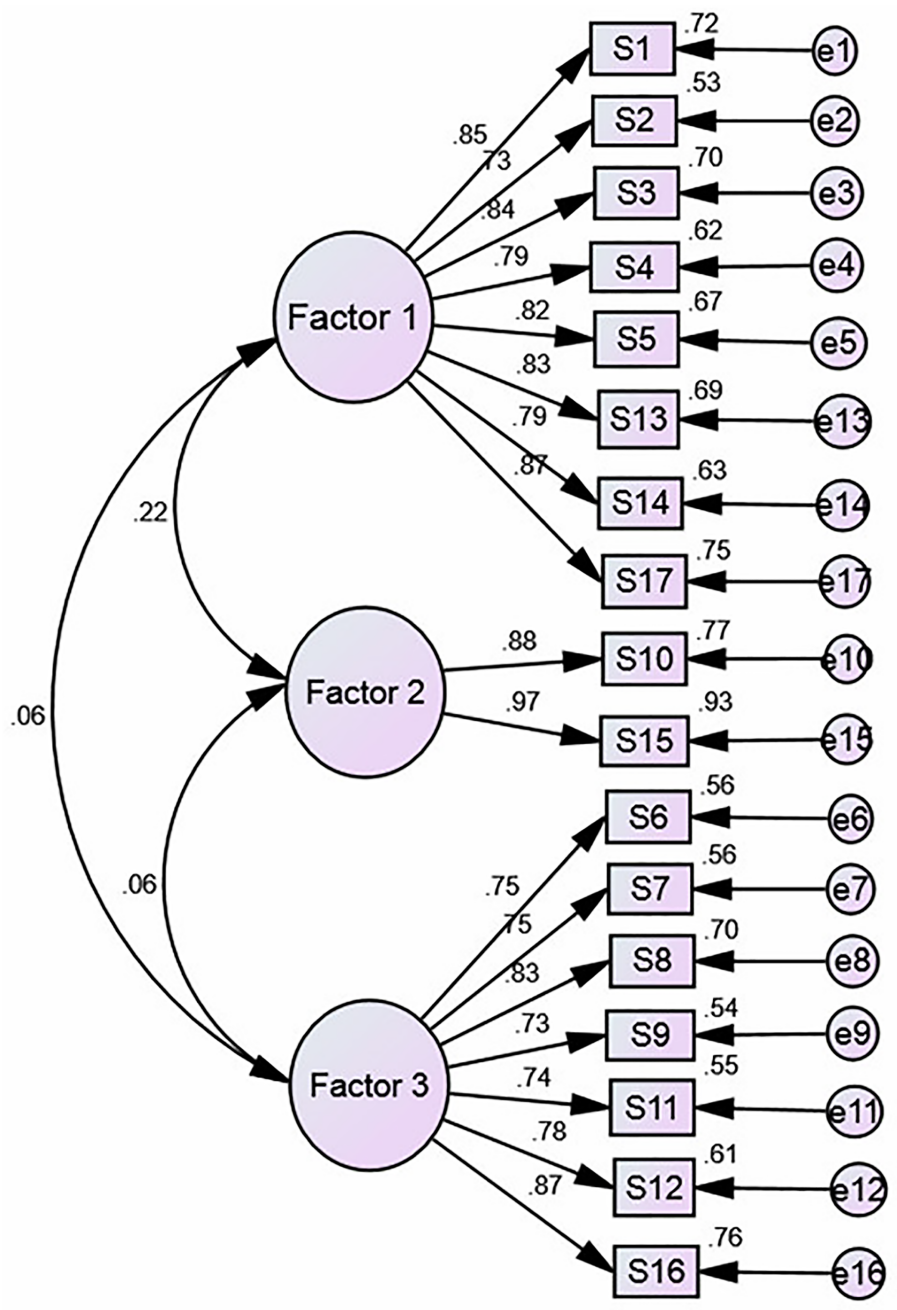
Standardized three-factor structural model of the SASE-CHI (n = 576). *Note*. SASE-CHI: the Chinese version of Self-care Ability Scale for the Elderly; Factor 1 (the repertoire, 8 items), Factor 2 (the environment, 2 items) and Factor 3 (the goals, 7 items); S1-S17: Item 1-Item 17, and each item is explained in Table 2. e1—e17: the measurement error of each observed variable to estimate latent variables.

**Fig 4:**
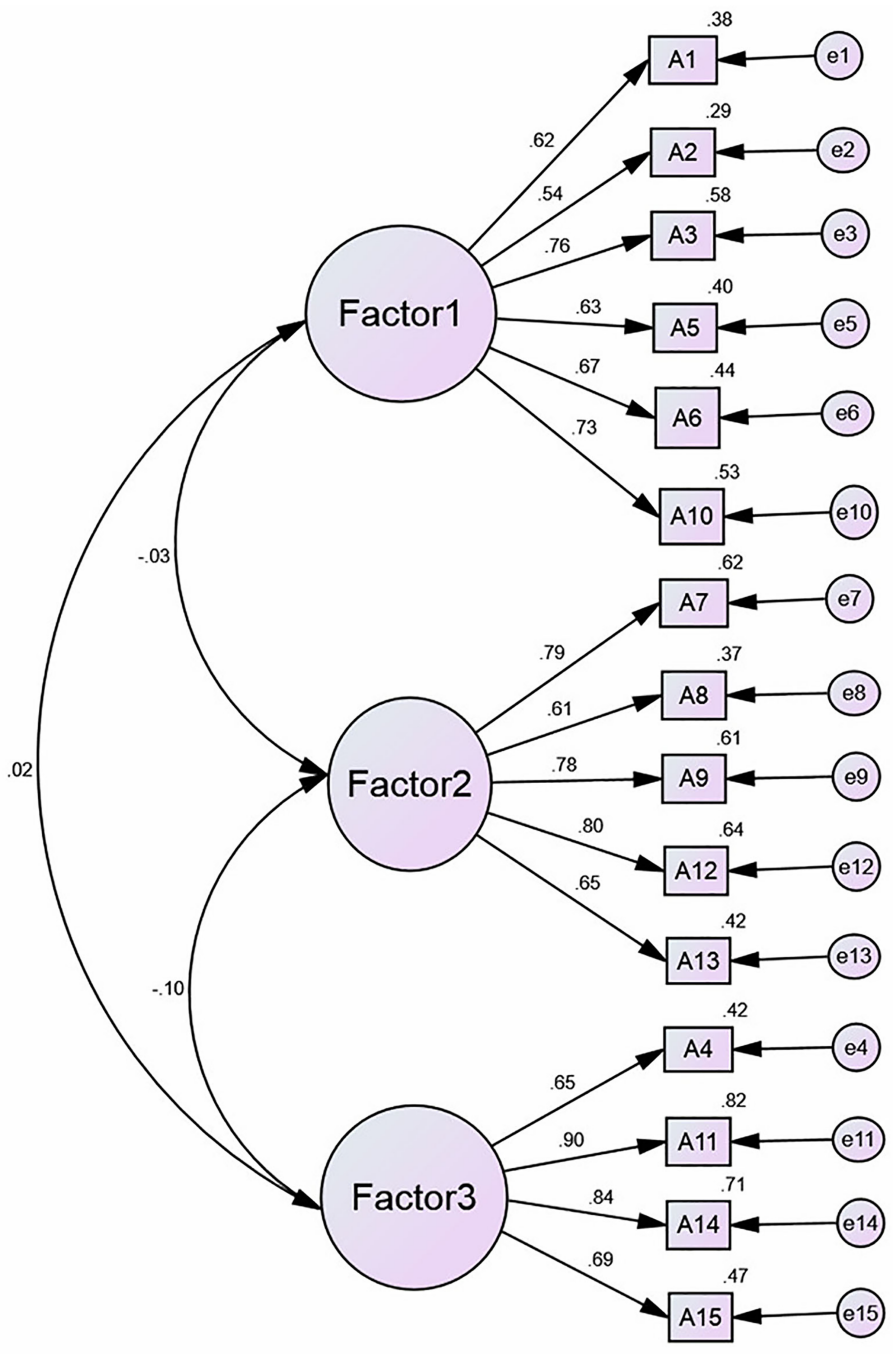
Standardized three-factor structural model of the ASAS-R-CHI (n = 576). *Note*. ASAS-R-CHI: the Chinese version of the Appraisal of Self-Care Agency Scale-Revised; Factor 1 (having power for self-care, 6 items), Factor 2 (developing power for self-care, 5 items) and Factor 3 (lacking power for self-care, 4 items); A1-A15: Item 1-Item 15, and each item is explained in Table 3. e1—e15: the measurement error of each observed variable to estimate latent variables.

The results of the EFA of SASE-CHI and ASAS-R-CHI indicated that the Kaiser–Meyer–Olkin (KMO) were 0.84 and 0.81, and Bartlett's Test of Sphericity was 3383.60 and 2,845.70, respectively, with statistical significance (*P*<0.01). Three factors were extracted with an eigenvalue greater than 1.00 of SASE-CHI (See Fig 1) and ASAS-R-CHI (See Fig 2) after principal components analysis and Varimax orthogonal rotation. The three extracted factors explained 72.65% and 64.93%, respectively, of the total variance of the two scales. The CFA of SASE-CHI and ASAS-R-CHI indicated that all measurements of the structural equation model were good fit (Table 4). Standardized three-factor structural model of SASE-CHI appears in Fig 3 and the standardized three-factor structural model of ASAS-R-CHI appears in Fig 4.

**Table 4:** The ASAS-R-CHI and SASE-CHI model fit measure (*n* = 576).

Structure factor model	CMIN/DF (*x*^2^/*df*)	GFI	AGFI	CFI	TLI	RMSEA	RMR	PLCODE
Three-factor model of ASAS-R-CHI	2.62	0.94	0.92	0.93	0.91	0.06	0.02	0.67
Three-factor model of SASE-CHI	2.60	0.95	0.93	0.94	0.93	0.04	0.01	0.76

*Note*. *x*^2^/*df*, chi-square/degree of freedom; GFI, goodness of fitness index; AGFI, adjusted goodness of fit index; CFI, comparative fit index; TLI, Tucker Lewis index, RMSEA, root mean square error of approximation; RMR, root mean residual; PCLOSE, P-value for test of close fit.

#### Concurrent validity

The Pearson's rank correlation coefficients between the total score of SASE-CHI and ESCA and their factors showed that the two instruments were significantly correlated (*P*<0.01, *r* = 0.46 to 0.70). As well as ASAS-R-CHI and ESCA and their factors were significantly correlated (*P*<0.01, *r* = 0.39 to 0.67). See Table 5.

**Table 5:** The Pearson's rank correlation coefficients of three scales (*n* = 1152).

	ASAS-R-CHI	A-F1	A-F2	A-F3	SASE-CHI	S-F1	S-F2	S-F3	ESCA	E-F1	E-F2	E-F3	E-F4
ASAS-R-CHI	—												
A-F1	0.82^※^	—											
A-F2	0.72^※^	0.61^※^	—										
A-F3	0.54^※^	0.47^※^	0.35^※^	—									
SASE-CHI	0.65^※^	0.37^※^	0.41^※^	0.35^※^	—								
S-F1	0.52^※^	0.44^※^	0.54^※^	0.67^※^	0.77^※^	—							
S-F2	0.48^※^	0.51^※^	0.45^※^	0.33^※^	0.56^※^	0.65^※^	—						
S-F3	0.52^※^	0.62^※^	0.63^※^	0.44^※^	0.66^※^	0.69^※^	0.55^※^	—					
ESCA	0.62^※^	0.65^※^	0.65^※^	0.63^※^	0.70^※^	0.52^※^	0.49^※^	0.52^※^	—				
E-F1	0.53^※^	0.46^※^	0.55^※^	0.39^※^	0.47^※^	0.47^※^	0.48^※^	0.52^※^	0.61^※^	—			
E-F2	0.62^※^	0.67^※^	0.64^※^	0.52^※^	0.54^※^	0.47^※^	0.46^※^	0.51^※^	0.71^※^	0.73^※^	—		
E-F3	0.54^※^	0.45^※^	0.63^※^	0.67^※^	0.51^※^	0.52^※^	0.47^※^	0.48^※^	0.67^※^	0.53^※^	0.62^※^	—	
E-F4	0.52^※^	0.48^※^	0.55^※^	0.55^※^	0.47^※^	0.55^※^	0.57^※^	0.58^※^	0.80^※^	0.66^※^	0.58^※^	0.66^※^	—

SASE-CHI: the Chinese version of Self-care Ability Scale for the Elderly. S-F1, S-F2, and S-F3 are factor 1, factor 2 and factor 3 of SASE, respectively.

ASAS-R-CHI: the Chinese version of the revised form of the Appraisal of Self-care Agency Scale. A-F1, A-F2, and A-F3 are factor 1, factor 2 and factor 3 of ASAS-R-CHI, respectively.

ESCA: the Exercise of Self-care Agency scale. E-F1, E-F2, E-F3, and E-F4 are factor 1, factor 2, factor 3 and factor 4 of ESCA, respectively.

^※^P<0.01.

#### Sensitivity and specificity

**Fig 5:**
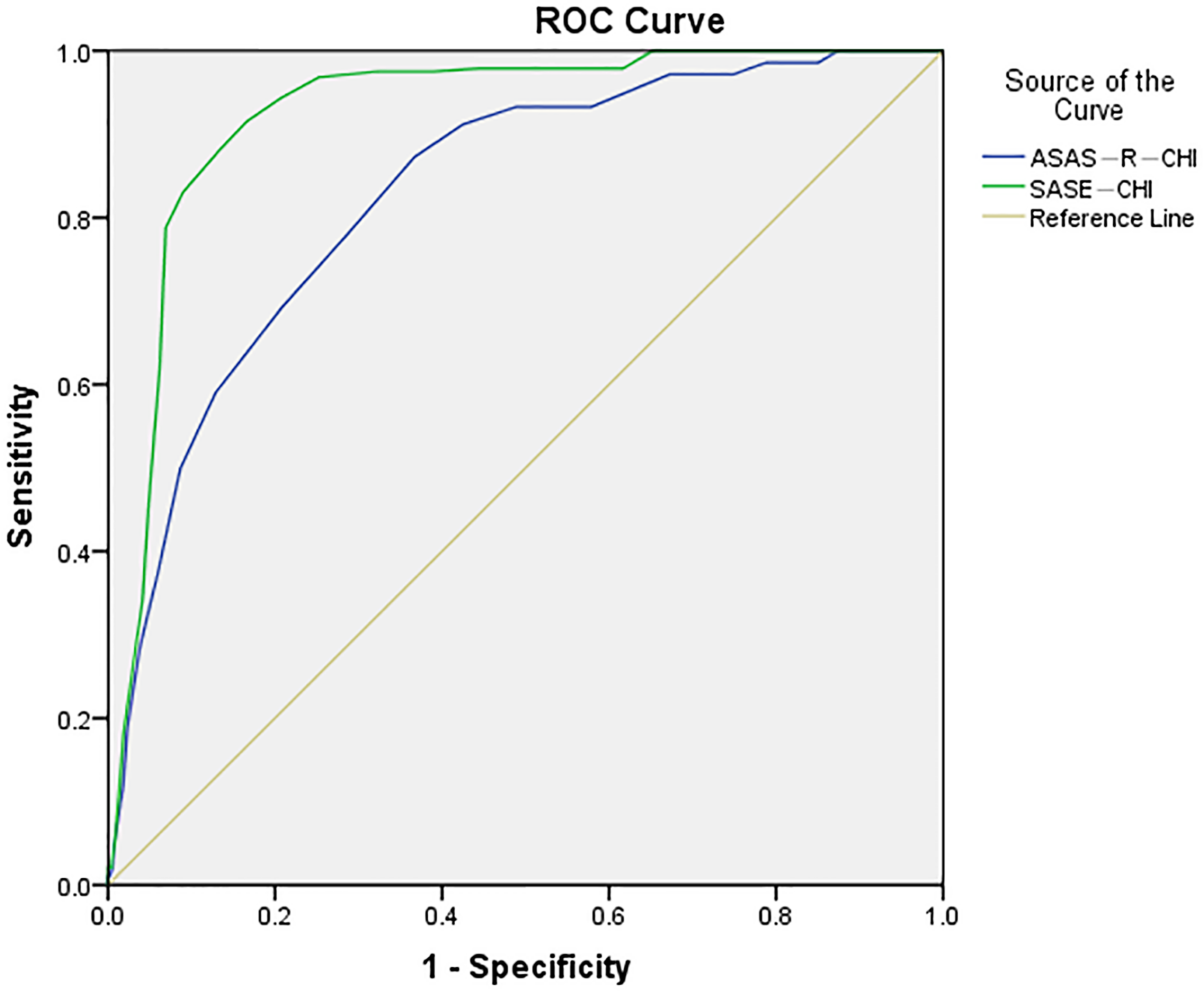
The receiver operator characteristic (ROC) curve of the SASE-CHI and ASAS-R-CHI with ESCA as a criterion (*n* = 1152). *Note*. SASE-CHI: the Chinese version of Self-care Ability Scale for the Elderly. ASAS-R-CHI: the Chinese version of the revised form of the Appraisal of Self-care Agency Scale. ESCA: the Exercise of Self-care Agency scale.

The result of the ROC curves showed that the area under the ROC curve of SASE-CHI for the optimal cut-point was 0.93 (95% CI, 0.91–0.94), and the area under the ROC curve of ASAS-R-CHI for the optimal cut-point was 0.83 (95% CI, 0.80–0.86). The optimal cut-off point of ASAS-R-CHI was assessed to be ≤56 (indicating lower level of self-care ability) and the optimal cut-off point of SASE-CHI was assessed to be ≤66 (indicating lower level of self-care ability), based on the values for sensitivity, specificity, PPV, NPV, Youden's index, and the ROC curves (Fig 5, Tables 6 and 7).

**Table 6:** Sensitivity and specificity for SASE-CHI with ESCA as a criterion (*n* = 1152).

Cut-off points	Sensitivity (%)	Specificity (%)	Youden's index	PPV (%)	NPV (%)
56.5	100	34.8	0.348	82.15	100
57.5	97.9	38.3	0.362	82.64	85.87
58.5	97.9	42.8	0.407	83.70	87.17
59.5	97.9	48.7	0.466	85.13	88.55
60.5	97.9	55.6	0.535	86.87	89.82
61.5	97.5	61.1	0.586	88.26	96.07
62.5	97.5	67.8	0.653	90.08	96.44
63.5	96.8	74.8	0.716	92.01	88.63
64.5	94.3	79.3	0.736	93.18	82.26
65.5	91.5	83.4	0.749	94.30	76.58
66.5	88.0	86.8	0.748	95.24	70.68
67.5	83.0	91.0	0.740	96.51	64.08
68.5	78.8	93.1	0.719	86.59	59.41
69.5	62.2	93.8	0.560	96.78	71.28
70.5	45.2	95.2	0.404	96.58	36.67
71.5	34.3	95.9	0.302	96.16	59.34
72.5	18.0	98.2	0.162	96.77	39.92
73.5	11.7	98.6	0.103	96.16	27.13
74.5	4.9	99.2	0.041	96.05	25.80
75.5	2.8	99.5	0.023	94.38	25.44
76.5	2.1	99.5	0.016	96.25	25.31
78.0	2.1	100	0.021	100	25.40

SASE-CHI: the Chinese version of Self-care Ability Scale for the Elderly.

ESCA: the Exercise of Self-care Agency scale

**Table 7:** Sensitivity and specificity for ASAS-R-CHI with ESCA as a criterion (*n* = 1152).

cut-off points	Sensitivity (%)	Specificity (%)	Youden's index	PPV (%)	NPV (%)
47.5	100	12.5	0.125	77.42	100
48.5	98.6	15	0.136	77.68	78.13
49.5	98.6	21.2	0.198	78.96	85.71
50.5	97.2	25.2	0.224	79.59	75.00
51.5	97.2	32.8	0.300	81.27	79.61
52.5	93.3	42.1	0.354	82.86	67.68
53.5	93.3	51.1	0.444	85.13	68.53
54.5	91.2	57.5	0.487	86.55	68.53
55.5	87.3	63.3	0.506	87.71	62.43
56.5	77.7	71.6	0.493	89.74	51.70
57.5	69.3	79.2	0.485	90.91	46.23
58.5	59.0	87.1	0.461	89.52	37.21
59.5	49.8	91.4	0.412	94.56	37.77
60.5	37.5	94	0.315	94.94	33.39
61.5	28.3	96.2	0.245	94.95	30.61
62.5	18.7	97.7	0.164	96.06	28.60
63.5	12.0	98.2	0.102	95.24	27.11
64.5	4.6	99.3	0.039	99.50	25.76
65.5	2.1	99.4	0.015	91.30	25.29
66.5	1.1	99.8	0.009	94.29	50.23
68.5	1.1	100	0.011	100	75.21

ASAS-R-CHI: the Chinese version of the revised form of the Appraisal of Self-care Agency Scale. ESCA: the Exercise of Self-care Agency scale

## Discussion

The participates in the sample came from the community and hospitals, with aspects such as age, gender, occupation, and education background widely distributed. Thus, the sample had a good representation, indicating a heterogeneous sample. The numbers of low educated and smokers among the participants made it clear that the Chinese elderly in Jinzhou had a relatively low education and relatively poor living habits. This may be due to the low-level economy and society when they were young [38]. Using ESCA as an assessment instrument, the overall level of self-care of the older adults in Jinzhou was low, and the reason might be the failure in physiological function which is leading to the increase of chronic diseases (650 older persons with chronic diseases) and thus the reduction in self-care [37].

The values of Cronbach's α, Guttman Split-Coefficients, and Cronbach's α of each factor of the SASE-CHI ad ASAS-R-CHI were all within the recommended standard (>0.70) [39]. The obtained item-to-total correlations were in agreement with the recommended standard [40]. Thus, the results showed that the two scales have good homogeneity [39]. The correlation coefficients of the test-retest ICCs of SASE-CHI and ASAS-R-CHI evidenced that the two instruments are stable. All these analyses showed that SASE-CHI and ASAS-R-CHI are credible scales with a higher level of consistency and agreement compared to other studies of SASE and ASAS-R [2,6,11,15–17,24,27], and SASE-CHI is slightly stronger than ASAS-R-CHI in the present study.

The CVI of ASAS-R-CHI and SASE-CHI exceeded 0.90, demonstrating a good content validity [41], and the CVI of ASAS-R-CHI was slightly larger than the CVI of SASE-CHI. The Pearson's rank correlation coefficients between SASE-CHI and ESCA ranged from 0.46 to 0.70, and the values between ASAS-R-CHI and ESCA ranged from 0.39 to 0.67, as measures of concurrent validity. All correlations were significantly correlated, but the correlation coefficient between SASE-CHI and ESCA was weaker compared to the correlation coefficient between ASAS-R-CHI and ESCA.

Construct validity tested by EFA revealed three factors with an eigenvalue greater than 1.00 of SASE-CHI: the repertoire, 8 items; the environment, 2 items; and the goals, 7 items. The findings are consistent with Söderhamn et al. [16] and Gao et al. [28]. The EFA of ASAS-R-CHI also revealed three factors with an eigenvalue greater than 1.00: having power for self-care, 6 items; developing power for self-care, 5 items; and lacking power for self-care, 4 items. This is consistent with Sousa et al. [11] and Guo et al. [27]. CFA was used to confirm the construct validity of the SASE-CHI and ASAS-R-CHI, and the results showed that the factor loadings and explained variances of the two self-care instruments were all strong and consistent with the EFA, with the three-factor structure and good model fit indexes, yielding a good construct validity of the two self-care scales. The CFA of SASE-CHI and ASAS-R-CHI in the present study yielded a similar factor structure to the two previous studies [27,28].

The results of the ROC curves showed that the area under the ROC curve of SASE-CHI was larger than ASAS-R-CHI in this study. The area under the curve can best represent the effect of the scale's detection results, and it should be between 0.5 and 1.0. The higher the value, the better the effect [42]. The reason that the effect of SASE-CHI was better than ASAS-R-CHI could be twofold: first, SASE-CHI is a scale, developed especially for the older adults, while ASAS-R-CHI is suitable for all adults. However, the present sample consisted of older adults. Second, SASE-CHI is a self-reported scale, but ASAS-R-CHI can be used as a self-reported scale or by health professionals for assessing self-care ability of their patients, and because of this, different assessment methods may lead to different results [42].

A cut-off point is not evaluated for the American version of ASAS-R-CHI or the Brazil version of ASAS-R [11,24]. Therefore, this study is the first to explore the cut-off point of ASAS-R-CHI. The cut-off point of the Swedish version of SASE was 69 [17], and 71 for the Norwegian version of SASE [6]. The cut-off point of the Chinese version of the SASE was lower than the cut-off points of the Swedish version and the Norwegian version of SASE. An explanation could be that China is a developing country, therefore, the overall standard of living of Chinese older adults is lower compared to older adults in European countries. Thereby, the self-care level is also slightly lower than that of older adults in European countries, and the cut-off point would be reduced naturally.

The ASAS-R-CHI, which can be used as a self-reported scale or for assessment by health professionals, is suitable for all adults. It is based on Orem’s self-care deficit theory, and it focuses the assessment of personal self-care skills [11,24]. Nevertheless, SASE-CHI, a self-reported scale particularly for older adults, is based on the theory of health and adaptation of Pörn. This theory focuses on the assessment of self-awareness of individuals to their environment, and self-care intentions and self-care skills in a certain environment [43]. A self-report instrument can assess the potential of self-care ability that others cannot evaluate, such as the sense of self-care responsibility and self-concept. However, because of strong subjectivity, the self-reported instrument may underestimate or overestimate the self-care ability of the individual. So, when using self-reported scales, health professionals should observe daily behaviors of the individuals. Assessment instruments are more objective, compared to the self-reported scales, but are likely to ignore the individuals' initiative, that underestimate their self-care ability [43,44]. Therefore, the instruments that can be used as both for self-reporting or assessment are more reasonable [43,44].

This study has tested and compared the reliability and validity, sensitivity and specificity of two self-care related instruments, providing a power for the development of self-care scales, as well as a help for nurses to choose the appropriate scale for identifying and assessing older adults’ self-care. The present study has some limitations, for example, the sample was limited to one city, and it may have an influence on the result, so, a more diverse sample should be included in follow-up studies. The one-to-one and face-to-face survey method resulted in a high rate of response. This can also be a limitation, since the reserved personality of Chinese people could lead them to not provide their true answers to an unfamiliar person. Therefore, the level of self-care ability or self-care maybe was affected by this circumstance.

## Conclusions

SASE-CHI and ASAS-R-CHI are homogenous and stable instruments for self-care evaluation of older adults in hospitals and communities in China. However, further studies to test the reliability, validity, sensitivity, and specificity in different geographical populations in China should be performed. The two self-care instruments are equal, but with their own characteristics. However, SASE-CHI is most suitable for older adults. Most important is that the user can choose the best instrument according to the specific situation.
